# Author Correction: Rapid fabrication of reinforced and cell-laden vascular grafts structurally inspired by human coronary arteries

**DOI:** 10.1038/s41467-019-11446-9

**Published:** 2019-07-31

**Authors:** Tamara L. Akentjew, Claudia Terraza, Cristian Suazo, Jekaterina Maksimcuka, Camila A. Wilkens, Francisco Vargas, Gabriela Zavala, Macarena Ocaña, Javier Enrione, Claudio M. García-Herrera, Loreto M. Valenzuela, Jonny J. Blaker, Maroun Khoury, Juan Pablo Acevedo

**Affiliations:** 1 Laboratory of Nano-Regenerative Medicine, Faculty of Medicine, Universidad de los Andes, San Carlos de Apoquindo 2200, Las Condes, Santiago, 7620001 Chile; 2 Cells for Cells, Avda. Plaza 2501, Las Condes, Santiago, 7620157 Chile; 3 Consorcio Regenero, Avda. Plaza 2501, Las Condes, Santiago, 7620157 Chile; 4 Department of Chemical and Bioprocess Engineering, School of Engineering, Pontificia Universidad Católica de Chile, Avda. Vicuña Mackenna 4860, Macul, Santiago, 7820436 Chile; 5 School of Materials, MSS Tower, The University of Manchester, Manchester, M13 9PL UK; 6 Departamento de Cirugía Vascular y Endovascular, Pontificia Universidad Católica de Chile, Avda. Libertador Bernando O’Higgins 340, Santiago, 8331150 Chile; 7 Biopolymer Research and Engineering Lab (BiopREL), School of Nutrition and Dietetics, Faculty of Medicine, Universidad de los Andes, Avda. Plaza 2501, Las Condes, Santiago, 7620157 Chile; 8 Departmento de Ingeniería Mecánica, Universidad de Santiago de Chile, Avda. Libertador Bernando O’Higgins 3363, Estación Central, Santiago, 9170022 Chile; 9 Institute for Biological and Medical Engineering, Schools of Engineering, Medicine and Biological Sciences, Pontificia Universidad Católica de Chile, Libertador Bernando O’Higgins 340, Macul, Santiago, 7820436 Chile; 10 Center of Nanotechnology Research and Advanced Materials “CIEN -UC”, Pontificia Universidad Católica de Chile, Avda. Libertador Bernando O’Higgins 340, Macul, Santiago, 7820436 Chile; 11 Bio-Active Materials Group, School of Materials, MSS Tower, The University of Manchester, Manchester, M13 9PL UK

**Keywords:** Cardiac device therapy, Tissue engineering

Correction to: *Nature Communications* 10.1038/s41467-019-11090-3, published online 15 July 2019.

The original version of this Article contained an error in the spelling of the author Jekaterina Maksimcuka, which was incorrectly given as Jekaterina Maksimuck. This has now been corrected in both the PDF and HTML versions of the Article.

Additionally, the original PDF version of this Article contained an error in Figure 6c in which a third stress-strain curve was erroneously shown. The correct version of Fig. 6c is:

which replaces the previous incorrect version:

This has been corrected in the PDF version of the Article. The HTML version was correct from the time of publication.

