# Supplementary material for: Rapid fabrication of reinforced and cell-laden vascular grafts structurally inspired by human coronary arteries
Source: Nat Commun. 2019 Jul 15;10:3098. doi: 10.1038/s41467-019-11090-3 (PMC6629634; doi:10.1038/s41467-019-11090-3)
Supplement: Supplementary file 1 — Supplementary Information [file 41467_2019_11090_MOESM1_ESM.pdf]

Supplementary Information

"Rapid fabrication of reinforced and cell-laden vascular grafts structurally inspired by human coronary arteries"

Akentjew et al.

Supplementary Methods

Sacrificial alginate scaffold and vascular graft lumen formation

In order to obtain a smooth inner cellularized layer after removal of the vascular graft from a polymer rod or nitinol-based rod, a thin coating of sacrificial alginate scaffold was first generated around the rod. This allowed a gentle removal of the supporting rod from the inside of the vascular graft. A sacrificial alginate lining was deposited coating on a 3D printed plastic mandrel of 2.5 mm in diameter using the custom-made CNC machine following a previously described protocol[1]. Briefly, a 2% (w/v) alginate solution was prepared by dissolving medium viscosity sodium alginate (A2033, Sigma, USA) in PBS 1X under continuous stirring at 60°C. Ionic crosslinking of alginate scaffold was conducted using a $CaCl_2$ bath, prepared by dissolving $CaCl_2$ in double deionized (dd)H_2O at a concentration of 5% (w/v), and maintained at 4°C. The sacrificial alginate scaffold was built after two subsequent dips into the alginate solution, and then submerged during 15 s in the $CaCl_2$ solution for crosslinking, then finally immersed and rinsed three times in PBS for 1 min.

Specific middle and outer layer fabrication

The middle and outer layers, which represent the media and adventitia layers of a natural artery, respectively, were fabricated by intercalating GEAL and PCL sub-layers. First, a thin sacrificial alginate scaffold was deposited around the plastic rod to allow a gentle removal of the cylindrical multi-layer construct after fabrication. For the middle PCL sub-layers, the SBS nozzle was oriented at -21° with respect to the

circumferential axis (Supplementary Fig. 1a). A complete spin-down-upward movement of the rod was first performed with the CNC machine while blowing PCL fibres for orientated deposition at an angle of +21°. Then a down-upward cycle while spinning the rod in the opposite direction was performed using the CNC machine in order to deposit the fibres at -21°. Subsequently, the formation of a concentric GEAL sub-layer was carried out by dipping the fiber-coated rod into the GEAL solution and kept there for 30 s in order to allow the GEAL solution to permeate through the fibers of the PCL sub-layer prior to photo-crosslinking. Subsequently, the previously described process of emergence from solution and photo-crosslinking was applied to stabilize the hydrogel within the graft sublayer. One graft sublayer consists of typically one PCL sublayer and one crosslinked GEAL sublayer. The same general methodology was used for the outer layer fabrication, changing the SBS nozzle angle to +67° and -67° (Supplementary Fig. 1b).

Preconditioning and Tensile Tests

Preconditioning and tensile test were made at different rates. For every tensile test, five preconditioning cycles were performed with a rate of 10 mm min^{-1}. In the case of middle and outer layer, tensile testing were performed using a rate of 10 mm min^{-1} (Supplementary Fig. 2a). However, in the case of the complete SDVG, a slower rate of 1 mm min^{-1} was used in order to compare to the coronary artery data (Supplementary Fig. 3b).

Clockwise and anticlockwise movement to incorporate waviness in the deposited PCL fibres

The deposition of wavy fibres on the rod was achieved by using a clockwise and anticlockwise movement. This movement correspond to a fully step in one direction and half a step in the opposite direction (Supplementary Fig. 3).

X-Ray computer tomography imaging

In order to analyze the macro/micro-structure of the full bio-inspired SDVG, some constructs were freeze-dried, and images were acquired using a Micro-CT (SkyScan 1272, Bruker-microCT, Kontich, Belgium) operated at 40 kV and 250 µA. Additionally, a completed SDVG graft was fabricated without GEAL sublayers, to study fibres porosity and fibres configuration, to prevent artefacts from freeze-dried GEAL masking this information

in X-ray computer tomography studies. This PCL fibre based SDVG was used to obtain images from high-resolution synchrotron X-ray tomography, performed at the Diamond-Manchester imaging branchline using the experimental facility I13-2 from Diamond Light Source[2]. A polychromatic filtered parallel-beam was used with a 0.33 µm effective pixel size. Over the 180° rotation, 3600 projections were collected at 0.05 s exposure time. The projections were tomographically reconstructed into a 3D volume using a software developed at Diamond Light Source[3]. The visualization package Avizo was used to produce the 3D images and interrogate construct porosity.

Burst pressure and suture retention strength

Burst pressure testing were performed under the same setting of pressurization testing and after the preconditioning step (see material and methods in the manuscript). After preconditioning, SDVG were subjected to continuous increasing of internal pressures at a rate of 100 mmHg min^{-1} until failure. Suture retention strength was performed according to instructions explained in the document ANSI/AAMI/ISO 7198:1998/2001. Briefly, a 5 cm length sample obtained from a longitudinal SDVG cut is mounted by clamping one end at the tensile machine, whereas the other end is sutured in one point at 2 mm from the free edge, in which a half loop formed by the suture is fixed to the other movable clamp. The pulling speed was set to 100 mm min^{-1}. Grams-force was recorded at the time of suture failure.

***In vivo* functionality of the encapsulated cells in bio-inspired SDVGs**

Six- to eight-week-old male BALB/c wild-type mice and C57BL/6 wild-type mice (skin allograft donors as positive controls) were obtained from the Jackson Laboratory (USA). Experiments were conducted at the Universidad de los Andes and Cells for Cells Animal Facility (Santiago, Chile) following institutional guidelines for the care and experimentation of laboratory animals, with Institutional Ethical Committee approval.

Acellular SDVGs were fabricated using GEAL solution without cells, whereas cellularized SDVGs were fabricated mixing the GEAL solution with BM-MSCs at a concentration of 10 million cells mL^{-1} for encapsulate, described above. The alginate used within the GEAL solution is a medical grade product with minimal endotoxin presence (#4200106, PRONOVA UP MVG, NovaMatrix, Norway). Subcutaneous implantation of

cylindrical sections of both acellular and cellularized grafts was performed through a dorsal incision on anesthetized mice with vaporized sevoflurane, and then sutured.

Skin grafting was performed as described previously[5], as immune reaction control. Briefly, tail skin (~ 1 cm^2) from C57BL/6 donors was transplanted onto the dorsal area of BALB/c. At 14-days post-surgery, graft-draining lymph nodes (dLNs) (mouse axillary lymph nodes) from graft recipients were obtained for further analysis described below. At 14 days, the SDVGs and dLNs from recipients were explanted for further analysis, described below.

***In vivo* cell functionality study of SDVGs by flow cytometry**

Draining lymph nodes (dLNs) obtained after surgery were processed and the dLN cells quantified and stained using anti-mouse CD4 (Clone RM4-4), CD25 (Clone PC61), CD62L (Clone MEL-14), CD44 (Clone IM7), CD19(Clone 6D5) (BioLegend, San Diego, CA, USA) and Foxp3(Clone FJK-16s, eBioscience), conjugated with different fluorochromes. Flow cytometry data acquisition was performed with a FACS Canto II cytometer using FACS Diva software (BD Biosciences, San Jose, CA). Data were analyzed using flowjo software (Tree Star, Canton, OH).

Supplementary Note 1

Results of immunosupressive activity of the encapsulated cells in the SDVG

Although results of cell viability analysis after SDVG fabrication are high, cell functionality could still be compromised by the manufacturing process. *In vivo* experiments were performed to evaluate whether encapsulated cells remain functional after the fabrication process. It is known that non-medical grade alginate contains endotoxins[6], therefore, implantation of SDVGs fabricated with a non-medical grade alginate in immunocompetent mice is expected to induce immunological activation and inflammatory response. On the other hand, bone marrow mesenchymal stem cells (BM-MSCs) are known to have immunomodulatory activity; therefore, it is expected that the immunoreaction in response to the endotoxins-laden SDVG is ameliorated by the activity of this cell type.

At day 14 post implantation of the acellular and endotoxin-laden SDVG, most of the mice exhibited issues with incision healing (Supplementary Fig. 9a). In contrast, mice with implanted endotoxin-laden SDVG containing encapsulated BM-MSCs showed apparent complete wound healing (Supplementary Fig. 9b).

Analysis of immunocompetent mice with dorsal subcutaneous implantations demonstrate that SDVGs with encapsulated endotoxins and without BM-MSCs induced a graft-derived inflammatory reaction, characterized for instance by a delayed graft incision healing (Supplementary Fig. 9a), an increased number of total cells isolated from graft-draining lymph nodes (dLNs) (mouse axillary and brachial lymph nodes), and an increased percentage of CD4+ memory T cells and B cells in dLNs compared to BM-MSCs cellularized graft (Supplementary Fig. 10 b, c, and d respectively). Whereas subcutaneously implanted SDVGs with encapsulated BM-MSCs, exhibited no signs of inflammation and the incision healed after 14 days (Supplementary Fig. 9b). Reduced cell numbers in dLNs, and an augmented percentage of activated $CD4^+$ T cells, $CD4^+$ naïve T cells, and $CD4^+$ regulatory T cells (Supplementary Fig. 10 e, f and g respectively) compared to implanted acellularized grafts was also found. These results, and considering the immunophenotypic data of allogeneic skin graft, known for conducting inflammation and immune rejection[7,8], suggests that an immunomodulation is being carried out by the viable and functional encapsulated BM-MSCs, mainly characterized by an immunotolerance of the endotoxin-laden graft[9], decrease in the number of dLN cells, decreased presence of B-cells[10] and increased presence of regulatory T cells in dNLs[11], all previously described as functions of BM-MSCs.

Supplementary Figures

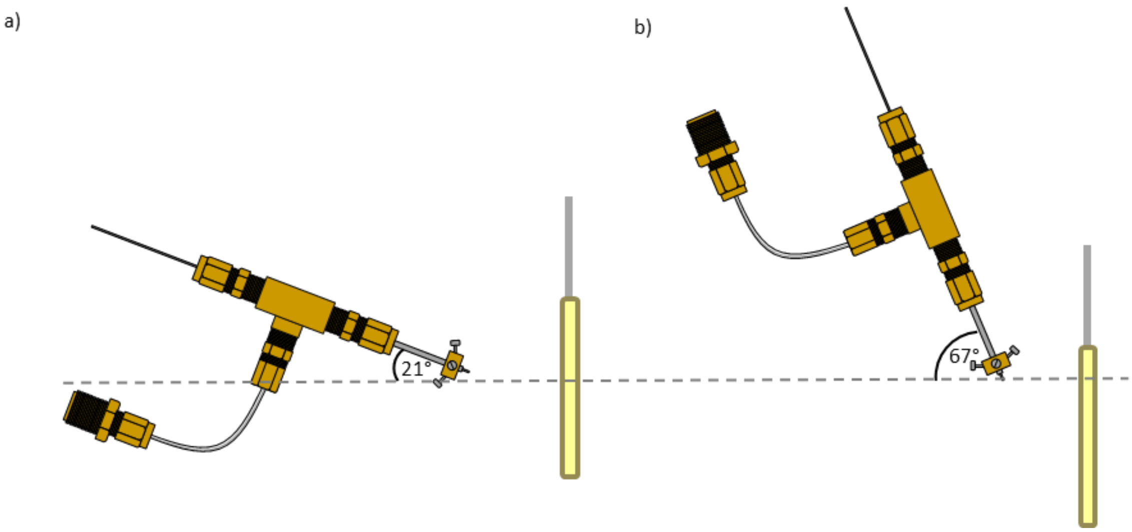

Supplementary Figure 1: SBS orientation for deposition of fibers in a) 21° and b) 67°

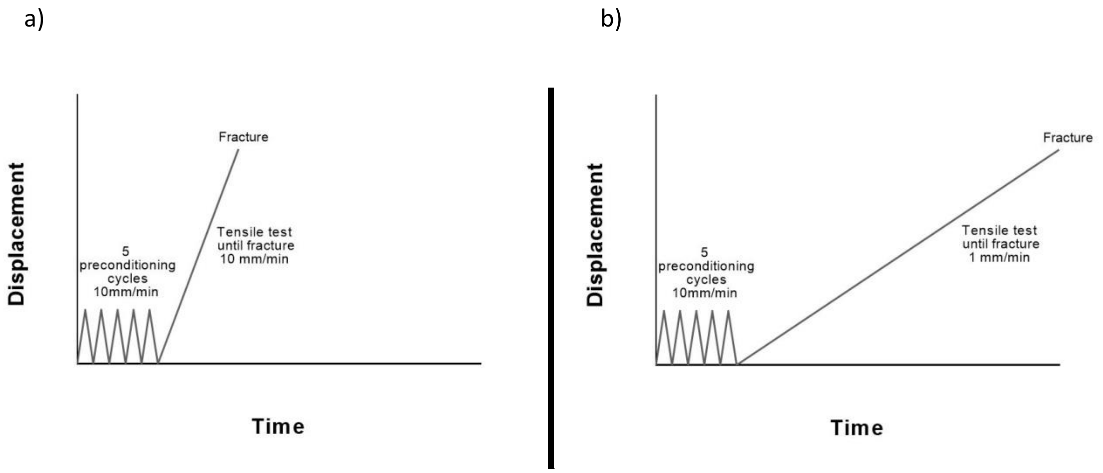

Supplementary Figure 2: Scheme of preconditioning cycles and tensile test rates of middle layer, outer layer and vascular graft. a) Middle and outer layers were preconditioned with five loading and unloading cycles at a constant rate of 10 mm min^{-1} and then tensile test was performed until fracture at the same constant rate of 10 mm min^{-1}. b) complete SDVGs were preconditioned with five loading and unloading cycles at a constant rate of 10 mm min^{-1} and then tensile test was performed until fracture at a constant rate of 1 mm min^{-1}.

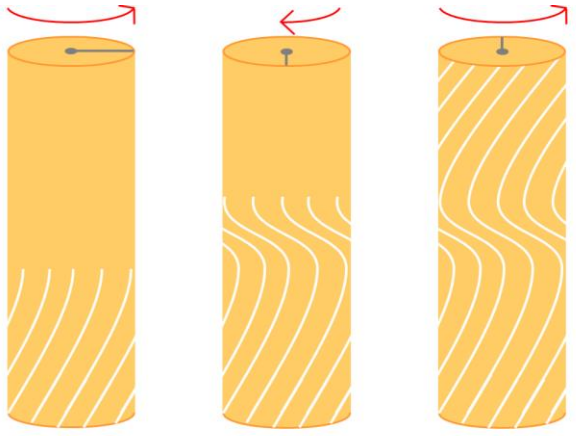

Supplementary Figure 3. Scheme of the clockwise and anticlockwise movement for fibres deposition including waviness.

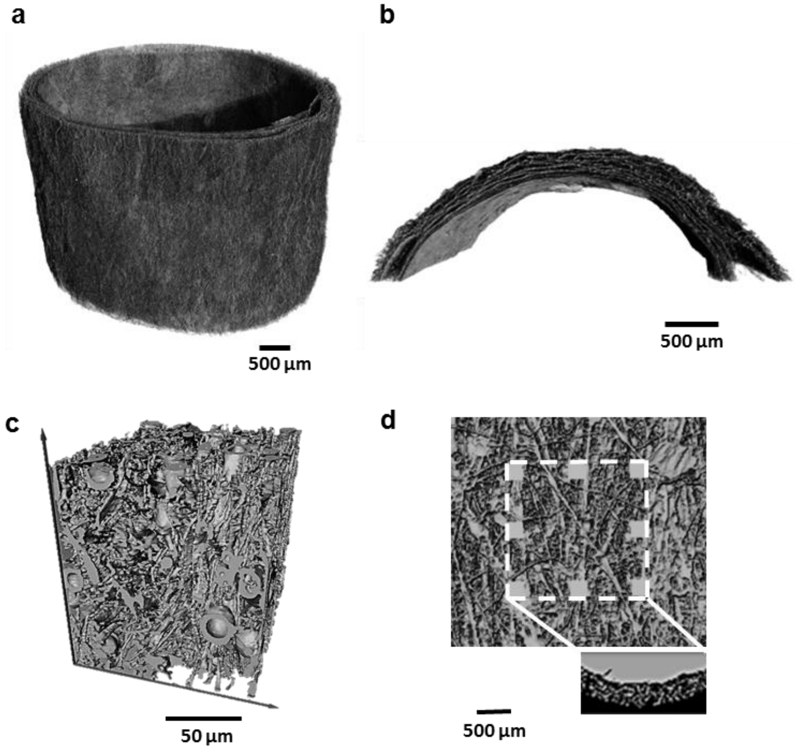

Supplementary Figure 4. Structural images of the SDVG through X-Ray computer tomography (CT). (a) CT of a lyophilized full SDVG and (b) the transversal cut. (c) Fibrillar and porous inspection of PCL sublayers in the SDVG fabricated without including the GEAL sublayer during fabrication. (d) Visualization of one section of the SDVGs fabricated without GEAL sublayers using package Avizo to produce the 3D image and porosity

analysis. A total of 5 sections of a defined 3D size (height x length x depth; 1700 µm x 1950 µm x 780 µm) were used to calculate SDVG porosity.

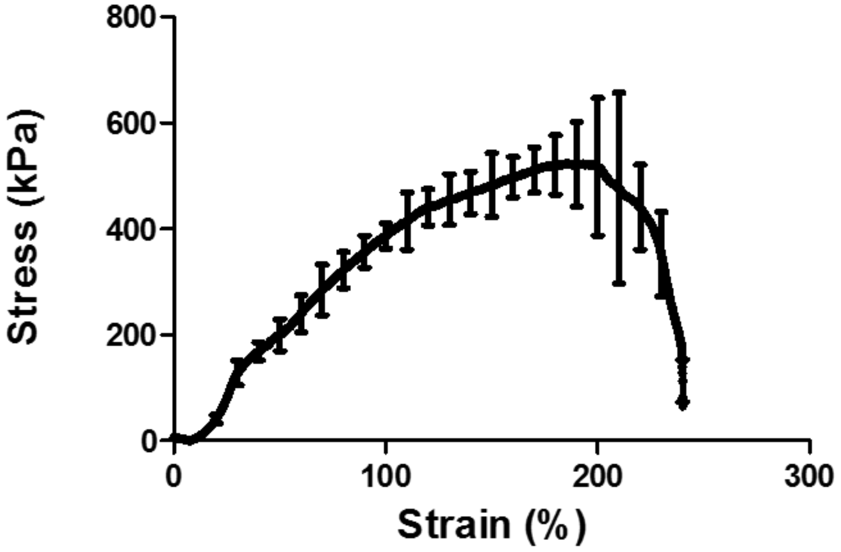

Supplementary Figure 5. Longitudinal stress-strain curve of the complete SDVG (9 PCL/GEAL sublayers) until failure (n = 3 independent experiments). Means and standard deviations are shown.

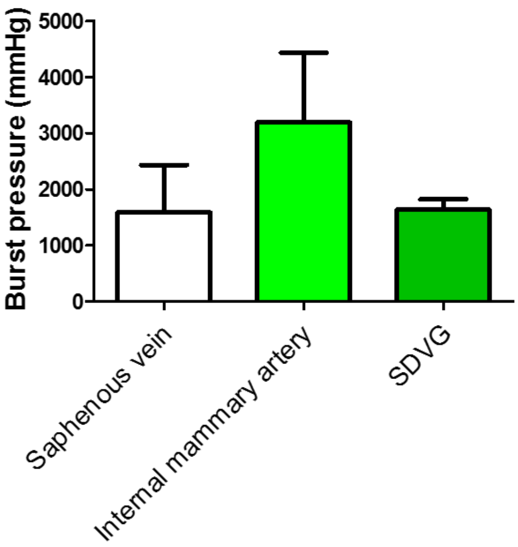

Supplementary Figure 6. Burst pressure comparison between obtained burst pressure for the new bio-inspired SDVG (n = 4 independent experiments) and reported data for saphenous vein and internal mammary artery[4] . Means and standard deviations are shown.

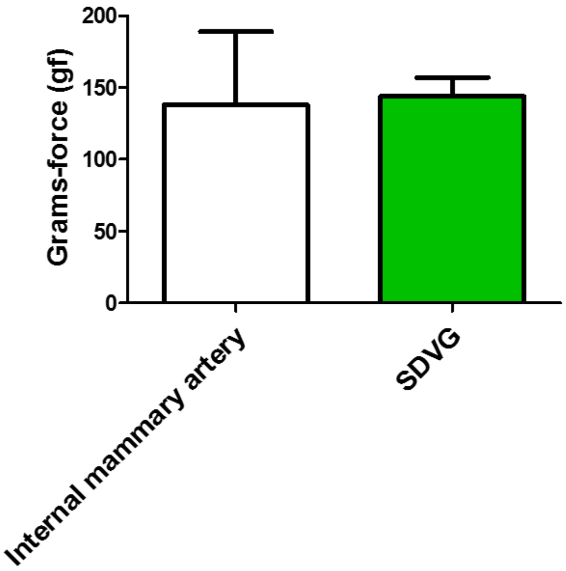

Supplementary Figure 7. Suture retention strength. Comparison between obtained suture retention strength for the new bio-inspired SDVG (n = 4 independent experiments) and reported data for the internal mammary artery[4]. Means and standard deviations are shown.

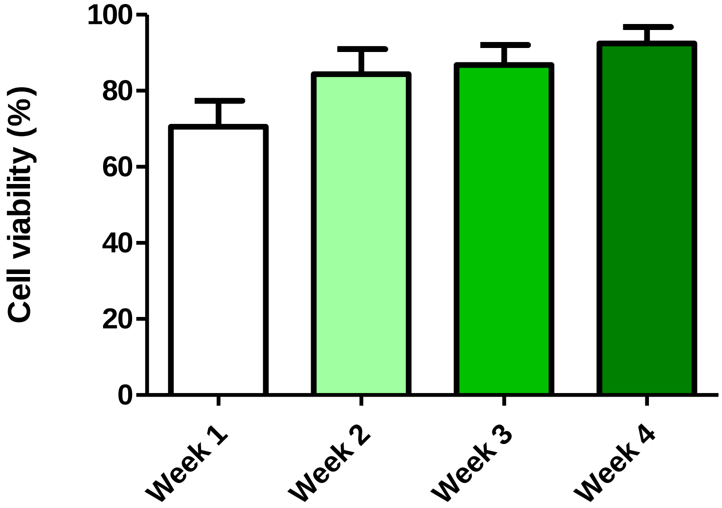

Supplementary Figure 8. Cell viability quantification of BM-MSCs encapsulated in the SDVG. Measurement were performed using a LIVE/DEAD assay (see materials and methods) and confocal microscopy over static cell cultured of SDVG at different time intervals; conducted in quadruplicate (mean and standard deviation shown).

a)

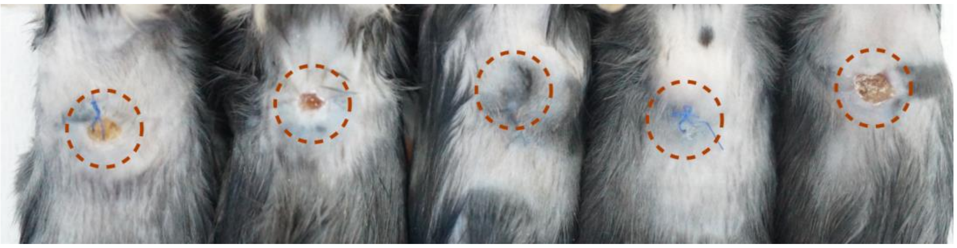

b)

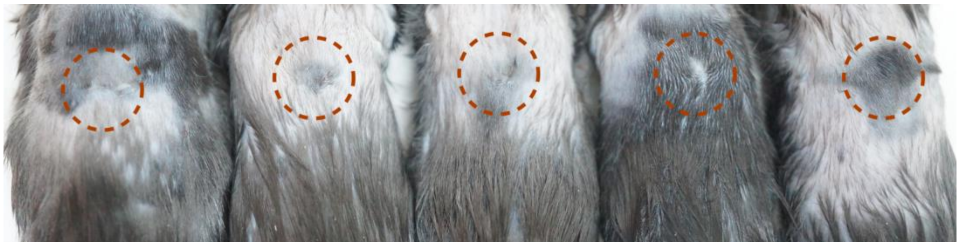

Supplementary Figure 9. Images from mice dorsal area for evaluating graft rejection. a) Acellular vascular grafts subcutaneously implanted. b) Cellularized vascular grafts subcutaneously implanted.

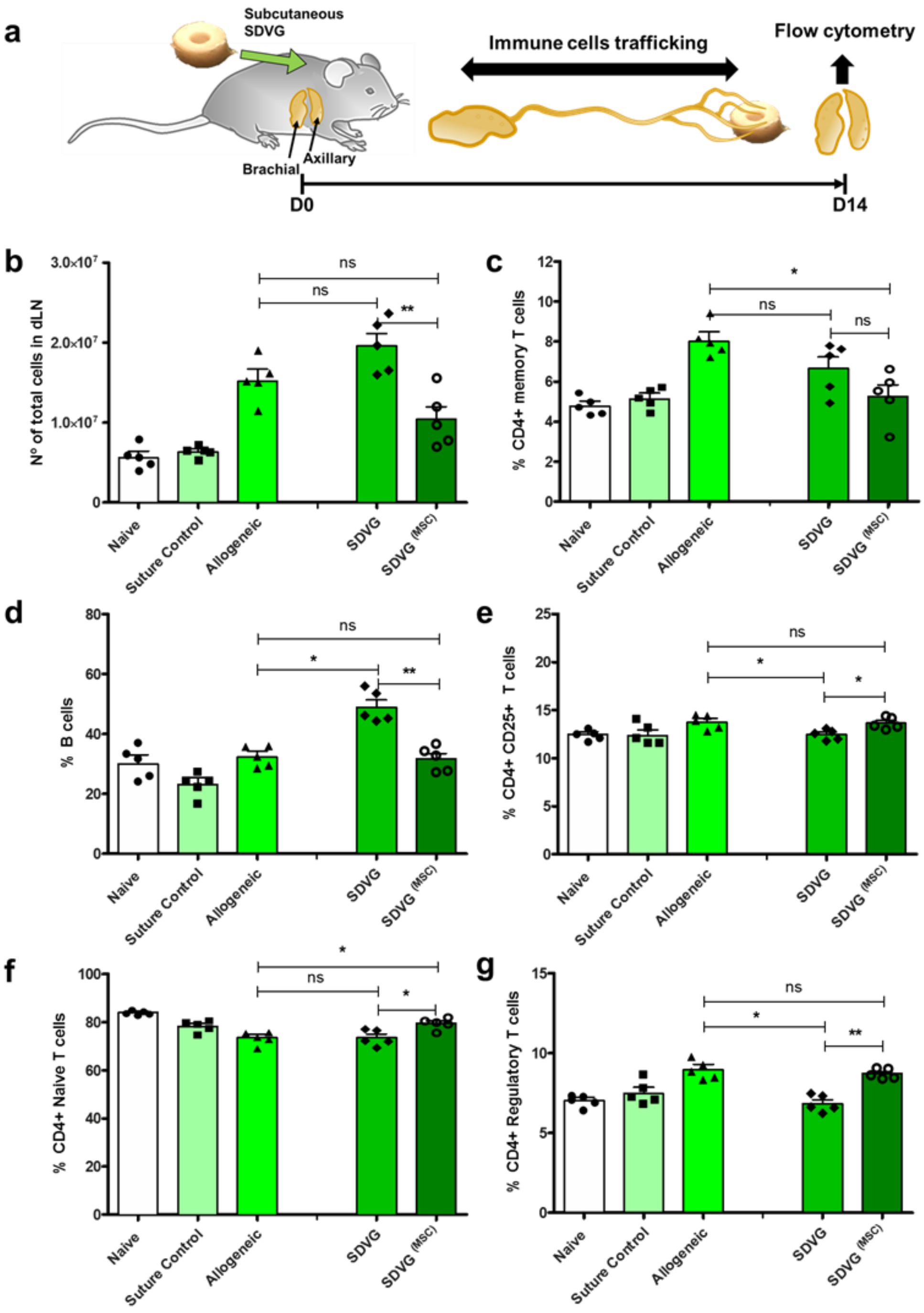

Supplementary Figure 10. Descriptions of immune results. (a) Schematic overview of the experimental immune challenge to assess the immunomodulatory function of laden BM-MSC in the SDVG. (b) Number of cells in dLN. (c) Frequency of CD62L$^-$ CD44$^+$ cells within CD3$^+$ CD4$^+$ cell population, corresponding to memory T Cells. (d) Frequency of CD19$^+$ cells in dLN, corresponding to B cells. (e) Frequency of CD25$^+$ cells within CD3$^+$ CD4$^+$ cell population, corresponding to activated T cells. (f) Frequency of CD62L$^+$ CD44$^-$ cells within CD3$^+$ CD4$^+$

cell population, corresponding to naïve T Cells. (g) Frequency of CD25^{high} Foxp3^{+} cells within CD3^{+} CD4^{+} cell population, corresponding to regulatory T cells. Naïve = non-operated control mice; Suture control = operated mice without any graft; Allogeneic = operated mice with allogenic skin graft. Error bars = standard error of the mean. * p<0.05; ** p<0.01; ns: non-significant. n=5 animals with 5 different SDVG fabrications. Statistical analysis was conducted using the Mann–Whitney U test.

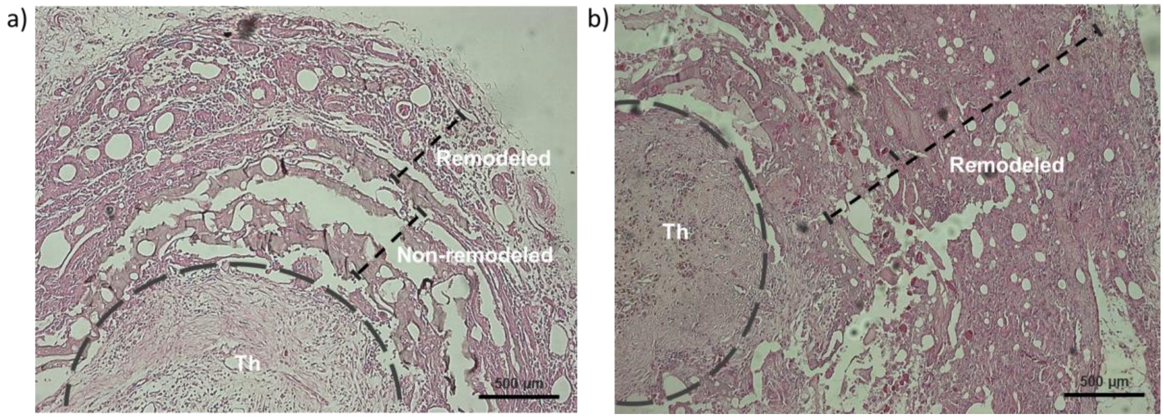

Supplementary Figure 11. H&E staining of a transversal section of explanted SDVG after 30 days of carotid arterial grafting in a rabbit model. a) Representative H&E staining of a acellularized SDVG. b) representative H&E staining of a cellularized (BM-MSCs) SDVG. Th = Luminal thrombus. Remodeled = Presence of cells and remodeled SDVG GEAL sublayers. Non-remodeled = Lack of cellularity and remodeling of GEAL sublayers.

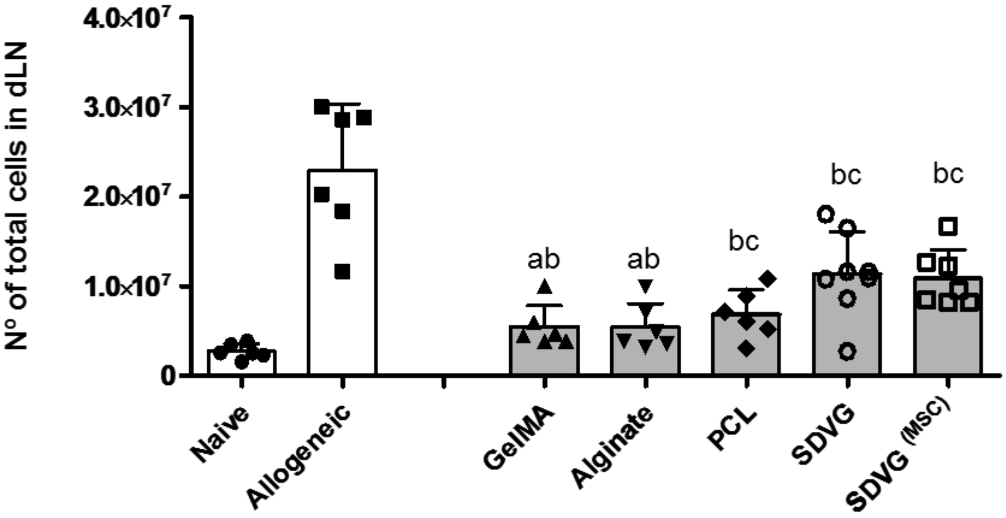

Supplementary Figure 12. Descriptions of the immune reaction in presence of the individual fabrication components of the SDVG and the fabricated SDVG with and without BM-MSCs. The alginate utilized for this experiment is a low endotoxin alginate. The number of cells in dLN (graft-draining lymph nodes, axillary and brachial) was quantified. Naïve = non-operated control mice; Allogeneic = operated mice with allogenic skin graft. Error bars = standard error of the mean. a = $p<0.05$ between the comparing group and naïve group; b = $p<0.01$ between the comparing group and allogeneic group; c = $p<0.01$ between the comparison group and naïve group. n=6 animals. Statistical analysis was conducted using the Mann–Whitney U test.

Supplementary Tables

Supplementary Table 1.

Bio-inspired small diameter vascular graft (n=5) and human coronary artery (n=5, data obtained from Claes, E.[12] and van Andel, C. J. et al.[13] compliance (%C) (10^{-2} mmHg) at different pressure ranges and longitudinal pre-stretch during testing (e_z). Standard deviation is presented too (±). Statistical analysis was conducted using the Mann–Whitney U test.

e_z	10%			20%			25%		
Pressure range (mmHg)	50-90	80-120	110-150	50-90	80-120	110-150	50-90	80-120	110-150
Vascular graft	29.2 ± 8.4	6.3 ± 2.2	5.2 ± 0.7	30.2 ± 7.8	9.3 ± 1.2	5.8 ± 0.4	24 ± 4.8	12.9 ± 2.6	7.5 ± 1
Coronary artery	5.7 ± 0.9*	3.8 ± 0.3	2.2 ± 0.3**	7.9 ± 2.3*	4.9 ± 1.4	2.5 ± 0.9	6.6 ± 1.3**	3.5 ± 0.7**	2.3 ± 0.6**

*Statistical difference ($p<0.05$) compared with SVDG

**Statistical difference ($p<0.005$) compared with SDVG

Supplementary References

1 Wilkens, C. A. *et al.* Layer-by-layer approach for a uniformed fabrication of a cell patterned vessel-like construct. *Biofabrication* **9**, 015001 (2016).

2 Rau, C., Wagner, U., Pešić, Z. & De Fanis, A. Coherent imaging at the Diamond beamline I13. *physica status solidi (a)* **208**, 2522-2525 (2011).

3 Atwood, R. C., Bodey, A. J., Price, S. W., Basham, M. & Drakopoulos, M. A high-throughput system for high-quality tomographic reconstruction of large datasets at Diamond Light Source. *Philosophical Transactions of the Royal Society of London A: Mathematical, Physical and Engineering Sciences* **373**, 20140398 (2015).

4 Konig, G. *et al.* Mechanical properties of completely autologous human tissue engineered blood vessels compared to human saphenous vein and mammary artery. *Biomaterials* **30**, 1542-1550 (2009).

5 Campos-Mora, M. *et al.* Neuropilin-1+ regulatory T cells promote skin allograft survival and modulate effector CD4+ T cells phenotypic signature. *Immunology and cell biology* **93**, 113-119 (2015).

6 Dusseault, J. et al. Evaluation of alginate purification methods: effect on polyphenol, endotoxin, and protein contamination. *Journal of Biomedical Materials Research Part A* **76**, 243-251 (2006).

7 Contreras-Kallens, P. et al. Mesenchymal stem cells and their immunosuppressive role in transplantation tolerance. *Annals of the New York Academy of Sciences* **1417**, 35-56 (2018).

8 Terraza, C., Fuentes, R. & Pino-Lagos, K. IFN-γ and IL-33 modulate mesenchymal stem cells function targeting Th1/Th17 axis in a murine skin transplantation model. *Cytokine* **111**, 317-324 (2018).

9 Kadle, R. L. et al. Microenvironmental cues enhance mesenchymal stem cell-mediated immunomodulation and regulatory T-cell expansion. PloS one 13, e0193178 (2018).

10 Corcione, A. et al. Human mesenchymal stem cells modulate B-cell functions. *Blood* **107**, 367-372 (2006).

11 Marfia, G. et al. Mesenchymal stem cells: potential for therapy and treatment of chronic non-healing skin wounds. *Organogenesis* **11**, 183-206 (2015).

12 Claes, E. *Estudio mecánico de las arterias coronarias humanas y sus sustitutos vasculares*, Caminos, (2010).

13 van Andel, C. J., Pistecky, P. V. & Borst, C. Mechanical properties of porcine and human arteries: implications for coronary anastomotic connectors. *The Annals of thoracic surgery* **76**, 58-64 (2003).
