# Supplementary material for: Rapid fabrication of reinforced and cell-laden vascular grafts structurally inspired by human coronary arteries
Source: Nat Commun. 2019 Jul 15;10:3098. doi: 10.1038/s41467-019-11090-3 (PMC6629634; doi:10.1038/s41467-019-11090-3)
Supplement: Supplementary file 3 — Reporting Summary [file 41467_2019_11090_MOESM3_ESM.pdf]

nature research

Corresponding author(s): DBPR NCOMMS-17-14751C

Last updated by author(s): Jun 19, 2019

Reporting Summary

Nature Research wishes to improve the reproducibility of the work that we publish. This form provides structure for consistency and transparency in reporting. For further information on Nature Research policies, see Authors & Referees and the Editorial Policy Checklist.

Statistics

For all statistical analyses, confirm that the following items are present in the figure legend, table legend, main text, or Methods section.

n/a	Confirmed	
☐	☒	The exact sample size (*n*) for each experimental group/condition, given as a discrete number and unit of measurement
☐	☒	A statement on whether measurements were taken from distinct samples or whether the same sample was measured repeatedly
☐	☒	The statistical test(s) used AND whether they are one- or two-sided<br>*Only common tests should be described solely by name; describe more complex techniques in the Methods section.*
☒	☐	A description of all covariates tested
☒	☐	A description of any assumptions or corrections, such as tests of normality and adjustment for multiple comparisons
☐	☒	A full description of the statistical parameters including central tendency (e.g. means) or other basic estimates (e.g. regression coefficient) AND variation (e.g. standard deviation) or associated estimates of uncertainty (e.g. confidence intervals)
☐	☒	For null hypothesis testing, the test statistic (e.g. *F*, *t*, *r*) with confidence intervals, effect sizes, degrees of freedom and *P* value noted<br>*Give P values as exact values whenever suitable.*
☒	☐	For Bayesian analysis, information on the choice of priors and Markov chain Monte Carlo settings
☒	☐	For hierarchical and complex designs, identification of the appropriate level for tests and full reporting of outcomes
☒	☐	Estimates of effect sizes (e.g. Cohen's *d*, Pearson's *r*), indicating how they were calculated

Our web collection on statistics for biologists contains articles on many of the points above.

Software and code

Policy information about availability of computer code

Data collection	*Provide a description of all commercial, open source and custom code used to collect the data in this study, specifying the version used OR state that no software was used.*
Data analysis	*Provide a description of all commercial, open source and custom code used to analyse the data in this study, specifying the version used OR state that no software was used.*

For manuscripts utilizing custom algorithms or software that are central to the research but not yet described in published literature, software must be made available to editors/reviewers. We strongly encourage code deposition in a community repository (e.g. GitHub). See the Nature Research guidelines for submitting code & software for further information.

Data

Policy information about availability of data

All manuscripts must include a data availability statement. This statement should provide the following information, where applicable:

- Accession codes, unique identifiers, or web links for publicly available datasets
- A list of figures that have associated raw data
- A description of any restrictions on data availability

The data that support the findings of this study are available from the corresponding author upon reasonable request. Additionally, the data that are displayed in the graphs of Fig. 5c-e are available in figshare with the identifier doi:10.6084/m9.figshare.8298449, and the data displayed in the graphs of Fig. 6a-b and Fig. 6d-f are available in figshare with the identifier doi:10.6084/m9.figshare.8298482.

Field-specific reporting

Please select the one below that is the best fit for your research. If you are not sure, read the appropriate sections before making your selection.

☒ Life sciences ☐ Behavioural & social sciences ☐ Ecological, evolutionary & environmental sciences

For a reference copy of the document with all sections, see nature.com/documents/nr-reporting-summary-flat.pdf

Life sciences study design

All studies must disclose on these points even when the disclosure is negative.

Sample size	Sample size calculations were determine using the on-line resource from IACUC of Boston University (https://www.bu.edu/researchsupport/compliance/animal-care/working-with-animals/research/sample-sizecalculations-iacuc/)71, with a power level of 95% and estimated variance from previous publications. Specifically for compliance experiments, estimated variance was determined based on Claes, E.34 and van Andel, C. J. et al.35, whereas for the immunocompetent mice model was based on Campos-Mora, M. et al.72.
Data exclusions	No data were excluded from the analysis.
Replication	Experiments were performed with 2-6 independent experiments.
Randomization	Random allocation of animals and experimental samples per group were applied.
Blinding	All experimental outcomes were obtained after blinded experimental and quantitative analysis of samples results.

Reporting for specific materials, systems and methods

We require information from authors about some types of materials, experimental systems and methods used in many studies. Here, indicate whether each material, system or method listed is relevant to your study. If you are not sure if a list item applies to your research, read the appropriate section before selecting a response.

Materials & experimental systems

n/a	Involved in the study	
☐	☒	Antibodies
☐	☒	Eukaryotic cell lines
☒	☐	Palaeontology
☐	☒	Animals and other organisms
☐	☒	Human research participants
☒	☐	Clinical data

Methods

n/a	Involved in the study	
☒	☐	ChIP-seq
☐	☒	Flow cytometry
☒	☐	MRI-based neuroimaging

Antibodies

Antibodies used	anti-human antibodies CD42a (Clone ALMA. 16), CD63 (Clone H5C6) and CD62P (Clone AK-4) all from BD Bioscience. Anti-mouse CD4 (Clone RM4-4), CD25 (Clone PC61), CD62L (Clone MEL-14), CD44 (Clone IM7), CD19(Clone 6D5) (BioLegend, San Diego, CA, USA) and Foxp3(Clone FJK-16s, eBioscience)
Validation	Validated clones are specified and the provider company too.

Eukaryotic cell lines

Policy information about cell lines

Cell line source(s)	Human umbilical cord endothelial Cells (HUVEC) (ATCC® CRL1730™) and bone marrow derived mesenchymal stem Cells (BM-MSCs, expanded until passage 5) (#PT-2501, Lot# 0000423370, LONZA, USA)
Authentication	Authentication was provided by the companies.
Mycoplasma contamination	Cell cultures before experiments and cell storage, are routinely subjected to mycoplasma testing (MycoAlertTM Mycoplasma Detection Kit, Lonza) following provider instructions.
Commonly misidentified lines (See ICLAC register)	n/a

Animals and other organisms

Policy information about studies involving animals; ARRIVE guidelines recommended for reporting animal research

Laboratory animals	For artery implantation model, six female New Zealand white rabbits weighing ~4 kg each, at the Universidad de los Andes Animal Facility (Santiago, Chile) following the institutional guidelines for care and experimentation with laboratory animals, with Institutional Ethical Committee approval. For immune reaction studies, Six- to eight-week-old male BALB/c wild-type mice and C57BL/6 wild-type mice (skin allograft donors as positive controls) were obtained from the Jackson Laboratory (USA).
Wild animals	*Provide details on animals observed in or captured in the field; report species, sex and age where possible. Describe how animals were caught and transported and what happened to captive animals after the study (if killed, explain why and describe method; if released, say where and when) OR state that the study did not involve wild animals.*
Field-collected samples	*For laboratory work with field-collected samples, describe all relevant parameters such as housing, maintenance, temperature, photoperiod and end-of-experiment protocol OR state that the study did not involve samples collected from the field.*
Ethics oversight	*Identify the organization(s) that approved or provided guidance on the study protocol, OR state that no ethical approval or guidance was required and explain why not.*

Note that full information on the approval of the study protocol must also be provided in the manuscript.

Human research participants

Policy information about studies involving human research participants

Population characteristics	Blood samples (10 mL) from six healthy human volunteers were extracted in sodium citrate blood collection tubes (BD Vacutainer, 369714, Becton, Dickinson and Company, USA) after signing a written informed consent, previously approved by Institutional Ethical Committee. Volunteers were ramdomly selected within a population of Males and Females with ages between 25-36.
Recruitment	Volunteers were ramdomly selected within a population of Males and Females with ages between 25-36.
Ethics oversight	Written informed consent, and protocol were previously approved by Institutional Ethical Committee at the University of the Andes.

Note that full information on the approval of the study protocol must also be provided in the manuscript.

Flow Cytometry

Plots

Confirm that:

☒ The axis labels state the marker and fluorochrome used (e.g. CD4-FITC).

☒ The axis scales are clearly visible. Include numbers along axes only for bottom left plot of group (a 'group' is an analysis of identical markers).

☐ All plots are contour plots with outliers or pseudocolor plots.

☒ A numerical value for number of cells or percentage (with statistics) is provided.

Methodology

Sample preparation	For platelets activation experiments, platelets rich plasma (PRP) was collected for Flow Cytometry analysis. Platelets were stained for 20 minutes at room temperature in the dark, using anti-human antibodies CD42a (Clone ALMA. 16), CD63 (Clone H5C6) and CD62P (Clone AK-4) all from BD Bioscience, conjugated to the fluorochromes FITC, PE and APC, respectively. In the immunogenecity study, draining lymph nodes (dLNs) obtained after surgery were processed and the dLN cells quantified and stained using anti-mouse CD4 (Clone RM4-4), CD25 (Clone PC61), CD62L (Clone MEL-14), CD44 (Clone IM7), CD19(Clone 6D5) (BioLegend, San Diego, CA, USA) and Foxp3(Clone FJK-16s, eBioscience), conjugated with different fluorochromes.
Instrument	FACSCanto IITM using the FACS Diva software (Becton Dickinson, CA, USA)
Software	FlowJo software (Tree Star, Canton, OH)
Cell population abundance	Cell population abundance for every subpopulation of activated CD42a platelets are indicated in the manuscript (Figure 8a, 8b). Cell population abundance for every subpopulation of dNL are indicated in supplementary information (Supplementary Fig. 10 and 12).
Gating strategy	For each sample, in both platelet activation and immunogenicity studies, the initial population was gated based on their Forward versus Side scatter properties (FSC vs SSC). Then, singlets were selected (FSC-A vs FSC-H). For platelets activation experiments, CD42a+ cells were gated as platelets, and then, the expression of the markers CD62P and CD63 were used in order to evaluate the platelet activation. For lymphocyte population in the immunogenicity study, dead cells were discarded using the viability staining (FSC-A vs LIVE/DEAD Fixable Near-IR dead Cell Stain kit). In the live cell population, CD4+ T cells were gated, and the

expression of CD25 and Foxp3 was used to identify the T regulatory cell population. For the identification of the naive or memory T cells populations, the expression of CD44 versus CD62L was used.

☐ Tick this box to confirm that a figure exemplifying the gating strategy is provided in the Supplementary Information.
